# Brief mental health interventions in conflict and emergency settings: an overview of four Médecins Sans Frontières – France programs

**DOI:** 10.1186/1752-1505-7-23

**Published:** 2013-11-01

**Authors:** Matthew E Coldiron, Augusto E Llosa, Thomas Roederer, German Casas, Marie-Rose Moro

**Affiliations:** 1 Epicentre, 8 rue Saint-Sabin, 75011 Paris, France; 2 Médecins Sans Frontières, 8 rue Saint-Sabin, 75011 Paris, France; 3 Los Andes University, School of Medicine, Cr 7 N. 117-15, Bogota, Colombia; 4 Hôpital Cochin, Maison des adolescents, Université de Paris 5, 97 Boulevard de Port Royal, 75670 Paris cedex 14, France

**Keywords:** Disasters, Therapeutic consultations, PTSD, Refugees, War, Violence, Natural disasters, Disaster medicine

## Abstract

**Introduction:**

Mental health problems, particularly anxiety and mood disorders, are prevalent in the setting of humanitarian emergencies, both natural and man-made disasters. Evidence regarding best strategies for therapeutic interventions is sparse. Médecins Sans Frontières has been providing mental health services during emergencies for over two decades, and here we compare data from four programs.

**Program Overview:**

In China, 564 patients were followed for an average of 7 sessions after a major earthquake. The most common diagnoses were PTSD and other anxiety disorders. Between program entry and exit, the median global assessment of functioning increased from 65 to 80. At program entry, 58% were considered moderately, markedly or severely ill; a proportion which fell to 14% at program exit. In Colombia in the setting of chronic violence, 2411 patients were followed for a median of two sessions. Anxiety disorders and major depression were the most common diagnoses, and 76% of patients were moderately or severely ill at program entry. 91% had symptomatic improvement at program exit. In Gaza, 1357 patients were followed for a median of 9 sessions; a majority was under age 15. PTSD and other anxiety disorders were the most common diagnoses, and 91% were moderately or severely ill at entry. 89% had improved symptoms at program exit. In the West Bank, the 1478 patients had similar characteristics to those enrolled in Gaza. 88% were moderately or severely ill at entry; 88% had improved at exit.

**Discussion and evaluation:**

It was feasible to implement brief yet effective mental health interventions in a wide variety of humanitarian contexts – post-natural disaster, during acute violent conflict and during chronic violent conflict. The most common diagnoses were PTSD, other anxiety disorders and mood disorders. The use of local specially-trained counselors who were focused on coping skills and improving functionality over a brief time period, likely contributed to the symptomatic improvement seen in a large majority of patients across the four sites.

**Conclusions:**

Mental health is an essential part of a health care response to humanitarian emergencies. In a variety of settings, we show the positive results of brief interventions. Further research is needed to improve and evaluate mental health interventions in crises.

## Background

Mental health problems are common in humanitarian settings – both those caused by war and violence and those caused by natural disasters [1]. One of the most common diagnoses following armed conflict and natural disasters is post-traumatic stress disorder (PTSD) [2,3]. Nonetheless, other mood and anxiety disorders are also highly prevalent [2,4-6]. Severe mental health problems such as psychosis are less common, and often antecedent to humanitarian emergencies, but their management in times of conflict and disaster can be difficult [7].

Given the high burden of mental health problems, provision of mental health services is an important part of an integrated package of care during humanitarian crises [8]. Nonetheless, good evidence about the most effective treatment strategies for children and adults is sparse, and current guidelines are based largely on expert opinions [9-11]. Better evidence about the role of mental health care in humanitarian settings is needed [12]. However, carrying out clinical research in these settings can be challenging, partially because of weakened health systems and structures. Likewise, specific, targeted interventions may not be appropriate in all cultures and regions.

Since 1989, MSF France has provided psychological care to persons affected by violence and lacking medical support [13,14], implementing mental health care programs in response to emergencies in over 40 countries on five continents worldwide. Principles of psychological first aid are used. The psychotherapeutic work that is offered through MSF programs is based on Winnicott’s therapeutic consultations model for children that we have adapted for adults and for varied cultural contexts [15,16]. We encourage individuals (adults, or where appropriate, children) and families or dyads (mothers with their children) to externalize their emotions and fears, to share their traumas, depression and anxiety, and to cope with their stresses [16]. We take into account different social and cultural contexts, using local therapists or translators [17-19], and we are guided by principles of psychodynamic and interpersonal psychotherapy [18,20]. While the details of the intervention may change slightly depending on the context, the overall psychotherapeutic technique remains the same. Patients with complex mental health problems and those not in the target population of the various interventions are referred to existing local health structures designed for longer-term psychological and psychiatric care.

Given the paucity of published literature on the subject, the cross-cultural aspect of MSF mental health programming can provide important insights on responding to emergencies. Here we describe patients enrolled in 4 mental health programs in emergency contexts, their diagnoses and their clinical evolution. Some of the data have been published individually [6,21,22]. In the context of this synthesis, we aim to show the results of brief psychological interventions in diverse emergency contexts, from areas experiencing chronic and acute violence as well as major natural disasters. Anonymized patient data were entered into Epidata (Odense, Denmark) and analyzed with STATA 10.1 (College Station, Texas, USA). The program was authorized by local authorities and patients were informed about the use of data in research. Privacy and confidentiality of patients were ensured during the treatment and after the conduct of the analysis. This analysis met the criteria for review of program monitoring data and for exemption from the MSF Ethics Review Board.

### Program overview

Table 1 presents demographic data as well as information about length of care for patients enrolled in programs in all four sites described below.

**Table 1:** Patient characteristics in four MSF mental health programs

	**China (N = 564)**	**Colombia (N = 2411)**	**Gaza (N = 1357)**	**Nablus (N = 1478)**
Sex*				
F	388 *(68.8%)*	1624 *(67.6%)*	617 *(45.5%)*	868 *(58.8%)*
M	176 *(31.2%)*	780 *(32.4%)*	738 *(54.5%)*	608 *(41.2%)*
**Median age (IQR)**	41 *(14–58)*	33 *(15–46)*	13 *(9–24)*	15 *(7–36)*
**Median # of sessions (IQR)**	7 *(5–9)*	2 *(1–3)*	9 *(6–12)*	7 *(4–10)*
**Median length of therapy in weeks (IQR)**	7 *(5–10)*	3 *(1–6)*	12 *(8–16)*	12 *(7–17)*

*Missing values for 7 patients in Colombia, 2 patients in Gaza, and 2 patients in Nablus.

#### China

On 12 May 2008, a strong earthquake (7.9 on Richter scale) hit the Sichuan province of China, killing at least 100 000 people and leaving 5 million displaced. In the first days after the earthquake, MSF established a standard response in support of local and national emergency relief officials, including emergency mental health services. From 12 November 2008 until 15 August 2009, in conjunction with the Crisis Intervention Center of the Chinese Academy of Sciences, MSF offered psychological care in Wudu and Beichuan, sites of temporary housing for displaced persons, located approximately 100 km from the earthquake’s epicenter. Patients referred from local health care providers, as well as those detected in community screenings, were referred to psychological care centers managed by MSF, the Chinese Academy of Science and the Crisis Intervention Center in the temporary camps.

Ten local volunteer counselors provided psychological consultations under the supervision of a single expatriate clinical psychologist. The locally-trained volunteer counselors also benefitted from additional crisis response training provided by MSF, as well as individual and team meetings led by senior mental health staff and case review. Brief psychological evaluations were made on self-referred patients, as well as those who had been referred from school and community workers and health care providers. Patients diagnosed with a psychological condition for which psychotherapy was considered beneficial were enrolled in follow-up therapy.

Table 2 lists the most common primary diagnoses among the 564 patients enrolled in follow-up programs. A majority of patients were diagnosed with anxiety disorders, including PTSD and generalized anxiety disorder. Other common diagnoses included bereavement and adjustment disorder. Global assessment of functioning (GAF) and CGI were recorded at each visit. At first contact, median GAF was 65 (IQR 60–75) and 327 (58%) of patients were described as moderately, markedly, or severely ill on the CGI. At the time of last contact, median GAF was 80 (IQR 75–85) and 78 (14%) patients were described as moderately, markedly, or severely ill.

**Table 2:** Clinical diagnoses representing at least 5% of formal diagnoses made in four MSF mental health programs*

**China (N = 564)**		**Colombia (n = 2323)**		**Gaza (n = 1332)**		**Nablus (N = 1478)**	
**Condition**	**Number (%)**	**Condition**	**Number (%)**	**Condition**	**Number (%)**	**Condition**	**Number (%)**
PTSD	206 (36)	Anxiety disorders^†^	750 (32)	PTSD	659 (50)	Anxiety disorders^†^	333 (25)
Anxiety disorders^†^	146 (26)	MDD	423 (18)	Anxiety disorders^†^	247 (19)	PTSD	226 (17)
Bereavement	49 (9)	ASD	230 (10)	MDD	175 (13)	MDD	149 (11)
Adjustment disorder	32 (6)	Adjustment disorder	197 (9)	Enuresis	61 (5)	ASD	147 (11)
PTSD + MDD	27 (5)	PTSD	196 (8)			Distress, no disorder	126 (9)
MDD	26 (5)					Enuresis	111 (8)

*PTSD = Post-traumatic stress disorder; MDD = Major depressive disorder; ASD = Acute stress disorder.

^†^*Excluding ASD and PTSD.*

#### Colombia

Colombia has been affected by internal armed conflict for the last 40 years. Since 2002, MSF has provided mental health programming in the Department of Tolima, long a center of activity for different armed groups. The civilian population of this area has experienced multiple displacements, kidnappings, extortion, and repeated acts of armed violence. In this area, short-term psychological care was provided at a fixed urban site and in mobile clinics in rural areas where access to care was impeded because of the conflict.

At first contact, screening for PTSD, anxiety disorder, and depression was performed using a self-administered checklist of signs, symptoms, and feelings. Severity of symptoms was assessed for those admitted into follow-up programs by considering the number and intensity of the signs and symptoms of the disorder. Patients received either individual or group psychotherapeutic interventions. A full description of the methods used has been previously published [21].

Here we summarize the outcomes of the 2411 patients enrolled in individual or group psychotherapy between February 2005 and February 2008. Overall, 2054 patients (85.2%) received individual psychotherapy; the others received either group or dyad therapy, such as mother-and-child therapy for children under 3 years. Primary diagnoses were available for 2323 of enrolled patients. The most common clinical diagnoses were anxiety disorders (excluding PTSD and acute stress disorder) and depression. Acute stress disorder, adjustment disorder, and PTSD were also frequently diagnosed (Table 2). At time of enrollment, 1519 patients (64.2%) had moderate symptoms and 275 (11.6%) had severe symptoms. Patient default was common (1236, 51.7%). At the time of last contact, information about improvement or aggravation of symptom severity was available for 1685 patients (69.9%) originally enrolled. Of those, 154 (9.1%) had unchanged or worsened symptoms and 1531 (90.9%) had improved symptoms.

#### Gaza

Densely-populated and impoverished, the Gaza Strip has seen repeated waves of political tension and armed conflict for much of the last decade. The use of rockets and mortars has led to loss of property and deteriorating infrastructure, as well as many violent injuries and deaths. Living in the midst of chronic conflict, with ongoing fears of exacerbation, its residents have experienced large-scale trauma. MSF has provided medical care in Gaza since 1989, and its mental health program has aimed to provide support to civilian victims of conflict. Given concerns about the applicability of standard criteria and categories in this cross-cultural and post-traumatic background, focus groups with local professionals were conducted at the beginning of the program in Gaza and the West Bank to better understand the cultural implications of presenting complaints and symptoms.

At first contact, semi-structured interviews with standardized questionnaires led to an eventual DSM-IV-TR diagnosis, assigned by MSF psychologists and psychiatrists. The severity of disorder (mild, moderate, severe) was assessed at the initial visit and at the final visit. A full description of the methods used has been previously published [22].

Patients enrolled in Gaza were younger than those treated in China and Colombia; over half were children under 15 years. Among the 1357 patients whose mental health care began in an MSF clinic between January 2007 and July 2011, a formal diagnosis was available for 1332 patients. A large majority of patients were diagnosed with anxiety disorders; fully one-half were diagnosed with PTSD (Table 2). Depression and enuresis were also common. Severity at first contact was judged as moderate in 754 patients (55.6%) and as severe in 474 (34.9%). In total, only 133 patients (10.2%) defaulted on their treatment program. An assessment of symptom severity at program exit was available for 1256 patients: 141 (11.2%) had unchanged or worsened symptoms and 1115 (88.8%) had improved symptoms.

#### West Bank

The city of Nablus is one of the major urban centers of the West Bank. This part of the Occupied Palestinian Territories has also known political tension and violent conflict over the past decade, but with a lower level of acute violence than in Gaza. MSF has provided mental health care in the West Bank since 1994, opening a program in Nablus in 2004.

As in Gaza, referrals to the program were made from local health care practitioners as well as through community outreach efforts. All referred patients were screened and assigned a primary diagnosis. Those with mental illness related to the political tension and violence were enrolled in follow-up with MSF; patients not meeting these criteria were referred to appropriate health care providers.

Here we describe results of 1478 patients enrolled in the MSF mental health program between January 2007 and December 2011. Just as in the Gaza program, over half of all patients enrolled were children under 15. A formal diagnosis was available for 1355 patients. Like in Gaza, a variety of anxiety disorders and depression were the most common diagnoses (Table 2). On the other hand, the diagnosis of PTSD was made less frequently than in Gaza. As in Gaza, the diagnosis of enuresis was common, likely reflecting the younger age of enrolled patients. Severity at first contact was judged as moderate in 689 patients (46.7%) and severe in 601 (40.7%). As in Gaza, patient default was low – 286 patients (21.0%). An assessment of symptom severity at last contact was available for 1206 patients: 146 (10.8%) had unchanged or worsened symptoms and 1060 (87.9%) had improved symptoms.

## Discussion and evaluation

The strength of the current overview is that it shows a straightforward model of care that has been adapted for use in a wide variety of humanitarian settings – from Latin America to the Middle East to China. Furthermore, in these diverse settings – acute conflict, chronic conflict, and post-natural disaster – the overall patient-level results were extremely satisfactory, with marked improvement of functionality and/or symptom intensity seen throughout.

On the most basic level, several lessons can be taken away from this series of programs. Most importantly, it is possible to provide high-quality mental health care in the midst of humanitarian emergencies. While this is not necessarily a new observation, many previous reports of the effectiveness of psychotherapeutic interventions have been in the setting of clinical trials, under controlled settings, and with a larger infrastructure. The data described here are simply programmatic. On one hand, this is not ideal for drawing conclusions, but on the other hand, given the large number of patients across such different settings, the strength of the results described here – under actual program conditions – underscores the importance of including this type of programming in a standard emergency response.

The clinical improvement of patients provides several learning points. First, we believe that our model of care provision is well-adapted for responding to humanitarian emergencies. Non-professionals undergo intense, short-term training on providing coping skills and brief counseling sessions. One benefit of this model is that the counselors begin their training with a high level of language skills, and often cultural competency, relevant to the populations for whom they are caring. On the other hand, the use of non-professionals as counselors creates a burden for the supervising mental health professionals, both in terms of diagnostic accuracy as well as therapeutic follow-up. It also limits the number of clinical diagnoses that are able to be cared for in one program. Nonetheless, given that a large majority of our patients suffered from a limited number of mood and anxiety disorders, the training of counselors is made easier.

The measures of outcomes described here are rudimentary – often measured as “improved”, “unchanged”, or “worsened”, and did not always correlate to quantitative measures. At the same time, given the diversity of the social and political contexts, in the absence of standardized transcultural evaluation methods, this sort of simple measurement is actually quite powerful. Using standard checklists of symptoms, mental health professionals with a variety of backgrounds and non-professional counselors were able to make judgments about patient outcomes. In these humanitarian emergencies, the first goal of our mental health programs was to improve functionality and symptoms. This high rate of symptom-based and functional improvement was seen in all of the projects described here.

Despite efforts, given the wide variety of contexts reported here, it is clear that clinical diagnoses were not completely standardized, thus limiting specific analysis of outcome by clinical diagnosis. This would be interesting to consider, particularly for post-traumatic stress disorder and other anxiety disorders, which were the most common diagnoses encountered. While our results showed good levels of clinical improvement, it would also be interesting to see if rates of response differed by age. Given the variety of different therapeutic interventions used (individual, dyad, and group therapy), and without a more structured and consistent method of assessment across contexts, we are hesitant to draw conclusions.

Lastly, it would also be interesting to consider the costs of these different interventions. Given the overall rapid improvement of patients enrolled in our programs, quantifying the financial needs for program implementation, which we imagine to be comparatively small, might provide additional impetus for the inclusion of similar programs in other humanitarian emergencies.

## Conclusions

Standardization of data collection and evaluation methods would allow for greater comparability across different contexts. While patient care is rightfully prioritized in humanitarian settings, standardized monitoring of mental health diagnoses and outcomes would facilitate program evaluation. Additional rigorous assessments are still needed, but there appears to be an overall benefit to appropriately-targeted psychotherapy in war and other humanitarian crises [6,12,22,23].

The interventions we describe here were short-term. Implementation of longer-term psychological follow-up, particularly in areas affected by chronic violence, remains challenging. In our programs, patients needing ongoing psychiatric care were referred to local health care structures and other partner organizations. While beyond the scope of this overview, and indeed beyond the scope of MSF mental health interventions, it would be interesting to further assess the psychological needs of this group of patients.

Few conclusive studies assessing the effectiveness of mental health interventions in crises have been conducted. As a result, there is an ongoing gap in the current evidence base on individual components of mental health packages as well as best therapeutic strategies for specific diagnoses in low resource settings. One such study showed positive results in the treatment of PTSD in survivors of sexual violence and could be used as a model for research [24]. Additional examples of studies which could be conducted in crises should include outcome assessments as well as quasi-experimental designs where there is a built-in comparator and cross-culturally validated assessment tool. It is important to note that ethical concerns and the need to provide treatment may preclude the use of individual randomization.

Providing mental health care services should be part of the standard response to humanitarian emergencies. Our experience in vastly different settings shows that it is both feasible and beneficial for the patients. Our model, using a variety of mental health care providers – professionals and specially-trained local counselors – allowed us to care for a large number of patients in difficult contexts.

## Abbreviations

CGI: Clinical global impressions scale; GAF: Global assessment of functioning; IQR: Inter-quartile range; MSF: *Médecins Sans Frontières* (Doctors without borders); PTSD: Post-traumatic stress disorder.

## Competing interests

The authors declare that they have no competing interests.

## Authors’ contributions

MC drafted the manuscript. AL participated in study design and in drafting the manuscript. TR performed data analysis. GC and MM participated in study design, coordinated data collection and provided ongoing programmatic support. All authors read and approved the final manuscript.
